# Hedgehog Signalling in the Embryonic Mouse Thymus

**DOI:** 10.3390/jdb4030022

**Published:** 2016-07-16

**Authors:** Alessandro Barbarulo, Ching-In Lau, Konstantinos Mengrelis, Susan Ross, Anisha Solanki, José Ignacio Saldaña, Tessa Crompton

**Affiliations:** Immunobiology Section, UCL Institute of Child Health, London WC1N 1EH, UK; a.barbarulo@ucl.ac.uk (A.B.); chingin.lau@ucl.ac.uk (C.-I.L.); konstantinos.mengrelis.11@ucl.ac.uk (K.M.); susan.ross@ucl.ac.uk (S.R.); a.solanki.12@ucl.ac.uk (A.S.); j.saldana@ucl.ac.uk (J.I.S.)

**Keywords:** thymus, Hedgehog, Shh, Ihh, T cell development, thymic epithelial cell (TEC), Gli3, Gli2, Gli1

## Abstract

T cells develop in the thymus, which provides an essential environment for T cell fate specification, and for the differentiation of multipotent progenitor cells into major histocompatibility complex (MHC)-restricted, non-autoreactive T cells. Here we review the role of the Hedgehog signalling pathway in T cell development, thymic epithelial cell (TEC) development, and thymocyte–TEC cross-talk in the embryonic mouse thymus during the last week of gestation.

## 1. Introduction

T cells develop in the thymus, which provides an essential environment for T cell fate specification, and for the differentiation of multipotent progenitor cells into major histocompatibility complex (MHC)-restricted, non-autoreactive T cells. Here we review the role of the hedgehog (Hh) signalling pathway in T cell development, thymic epithelial cell (TEC) development, and thymocyte–TEC cross-talk in the embryonic thymus. 

**Figure 1:**
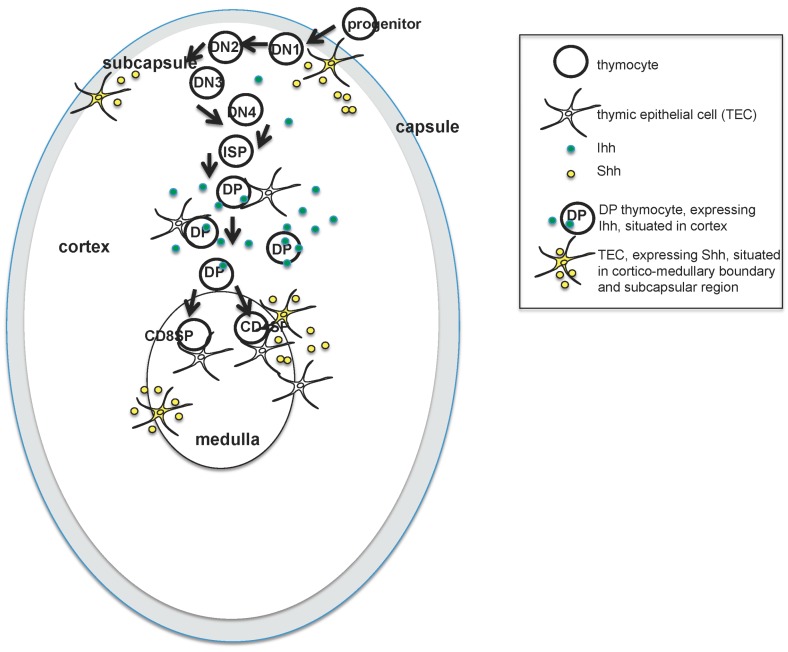
Hedgehog (Hh) expression in different microenvironments as thymocytes migrate through the embryonic thymus. The cartoon illustrates the stages of T cell development in the embryonic thymus, as developing thymocytes move through different thymus microenvironments and receive different amounts of Hh signal. Molecules of Indian hedgehog (Ihh, expressed by double positive (DP) thymocytes) are shown in green, and molecules of Sonic hedgehog (Shh, expressed by TEC in the sucbcapsular region and medulla) are shown in yellow. Progenitor cells first enter the embryonic thymus through the capsule on ~E12.5, and migrate towards the centre of the thymus as they differentiate. DP cells first appear on E16.5 and are located in the cortex. Mature single positive (SP) T cells and mature medullary TEC (mTEC) and cortical TEC (cTEC) populations are present by birth.

The thymus is an epithelial organ surrounded by a mesenchymal capsule. In the mouse embryo, it develops from the third pharyngeal pouches, which also gives rise to the parathyroids [1]. By embryonic day (E)12.5, the thymus and parathyroid have become distinct organs, and the thymus first starts to be seeded by haematopoietic progenitor cells that migrate from the foetal liver. Progenitor cells enter the thymus from the outside through the capsule, and migrate towards the center of the thymus as they differentiate (illustrated in Figure 1). This is in contrast to the adult thymus, which is seeded by progenitors that enter through blood vessels at the corticomedullary junction. 

During embryonic life, mature T cells are first produced on ~E18.5, which then start to leave the thymus and move to peripheral lymphoid organs by birth. In addition, TECs develop into two distinct populations—medullary(m) TEC and cortical(c) TEC—with different functions and locations within the thymus [2]. Haematopoietic progenitor cells are dependent on cTEC for T cell fate specification and positive selection of the T cell receptor repertoire (TCR), while mTEC are necessary for tolerance induction (negative selection of the TCR repertoire and differentiation of regulatory T cells) [3]. At the same time, TECs require signals from thymocytes for their development, and thymocyte–TEC cross talk in the embryonic thymus has been shown to be essential for establishment of the mature mTEC population and the architecture of the mature thymus, while its role in cTEC differentiation is less well-defined [2,4,5].

During T cell development, thymocytes pass through a series of stages that have been defined by the expression of cell surface markers as they migrate through the thymus [6] (see Figure 1). In brief, the earliest thymocytes—termed CD4−CD8− double negative (DN)—rearrange the TCRβ chain locus and express the pre-TCR in order to differentiate to the CD4+CD8+ double positive (DP) stage. They then rearrange the TCRα locus and must signal through the αβTCR to differentiate to mature CD4+ single positive (SP) or CD8+ SP T cell. The DN population has been further subdivided by cell surface expression of CD44 and CD25. The earliest DN1 populations are CD44+CD25−; these then acquire CD25 expression (DN2), lose CD44 expression (DN3), and finally become CD44−CD25− (DN4) cells before differentiating to DP cell, often via a CD8+ immature single positive (ISP) intermediate. 

The development of TEC in the embryonic thymus is less well understood, but both lineages of TEC originate from a common CD45−Epcam-1+CD40^low^CD205^low^ progenitor cell population [7,8]. As development proceeds, these cells gain greater intensity of CD40 and CD205 expression, resulting in transitional progenitors which have the potential to differentiate into two populations—cTEC (CD40^int^CD205^high^Ly51+) or mTEC (CD40^high^CD205^low^UEA-1+) [9,10]. 

## 2. Expression of Hedgehog (Hh) Proteins and Pathway Components in the Embryonic Thymus

There are three mammalian Hh proteins (Sonic Hh, Shh; Indian Hh, Ihh; and Desert Hh, Dhh), which share a common signalling pathway [11]. When Hh proteins bind to their cell surface receptor Patched1 (Ptch1), the inhibition of Ptch1 on the signal transducer molecule Smoothened (Smo) is relieved, and Smo transduces the Hh signal. At the end of the signalling pathway are the Gli family of transcription factors (Gli1, Gli2 and Gli3). Gli1 is an activator of transcription only, whereas Gli2 and Gli3 can be processed (cleavage and post-translational modification) to function as repressors of transcription in the absence of Hh and activators of transcription when the Hh signal is transduced [12,13]. Gli2 functions predominantly as a transcriptional activator in vivo and is required to initiate the Hh signal [14]. In general, Gli3 functions as a transcriptional repressor in vivo, and can act to repress expression of *Shh* by repression of an intermediate transcriptional activator [15]. Hh proteins are required for patterning, organogenesis, and cell fate determination in many tissues during development, including the foetal thymus [16,17,18,19,20].

**Figure 2:**
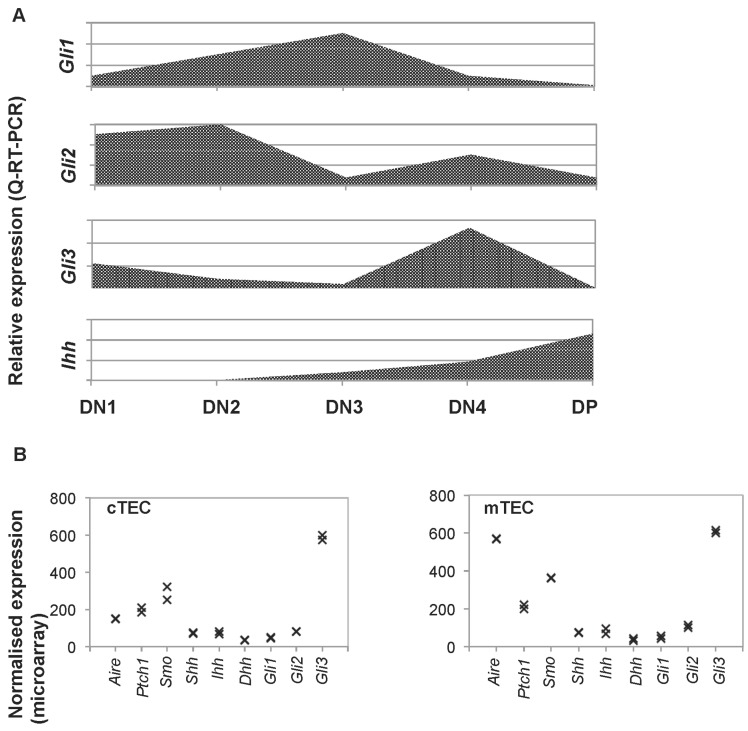
Expression of components of the Hh signalling pathway in embryonic thymocytes and TEC. (**A**) Graphs illustrate the relative expression of Gli1, Gli2, Gli3, and Ihh in sorted DN1, DN2, DN3, DN4, and DP thymocytes from E16.5 thymus, determined by quantitative reverse transcription polymerase chain reaction (QRT-PCR); (**B**) Plots show the normalised expression of Aire, Ptch1, Smo, Shh, Ihh, Dhh, Gli1, Gli2, and Gli3 by microarray analysis (GSE81433) from fluorescence activated cell sorting (Facs)-sorted cTEC (CD45−EpCam1+Ly51+UEA-1−, left-hand plot), and mTEC (CD45−EpCam1+Ly51−UEA-1+, right-hand plot); populations prepared from E14.5 WT (C57BL/6) foetal thymus organ culture (FTOC) after 7 days in culture. TEC were isolated as described [29].

Reverse transcription (RT)-PCR has shown that developing foetal thymocytes express the Gli transcription factors, Ptch1, Smo, and Ihh, but not Shh; however, TEC express Shh [21,22,23,24,25]. Quantitative (Q)RT-PCR from sorted thymocyte subsets on E16.5 showed that the Gli genes are differentially expressed during T cell development, with highest expression of Gli2 in the DN1 and DN2 populations, whereas Gli1 expression is highest in the DN3 population, and Gli3 in the DN4 population [21,23,24,25]. In contrast, Ihh expression is undetectable in the earliest DN thymocyte subsets, and is most highly expressed in the DP population (Figure 1 and Figure 2). Flow cytometry, RT-PCR and QRT-PCR have demonstrated that Smo is highly expressed in DN thymocytes, and down-regulated following pre-TCR signalling, whereas Ptch1 expression is maintained in DP cells [22]. Expression levels of Smo reduce with maturity in the DP population, but DP cells can transduce Hh signals [26]. 

Hh pathway components are also expressed by TEC in the embryonic thymus [21,22,27,28,29]. We carried out microarray analysis of gene expression in fluorescence activated cell sorting (Facs)-sorted cTEC and mTEC purified from E14.5 foetal thymus organ culture (FTOC) after 7 days in culture to allow maturation of both cTEC and mTEC populations (GSE81433). This analysis showed expression of Smo, Ptch1 and Hh genes, in addition to high expression of Gli3 (Figure 2B). Thus, in the foetal thymus, TECs express both the Hh proteins and the machinery to transduce the Hh signal. 

## 3. Regulation of T Cell Development by Hh Signalling

**Figure 3:**
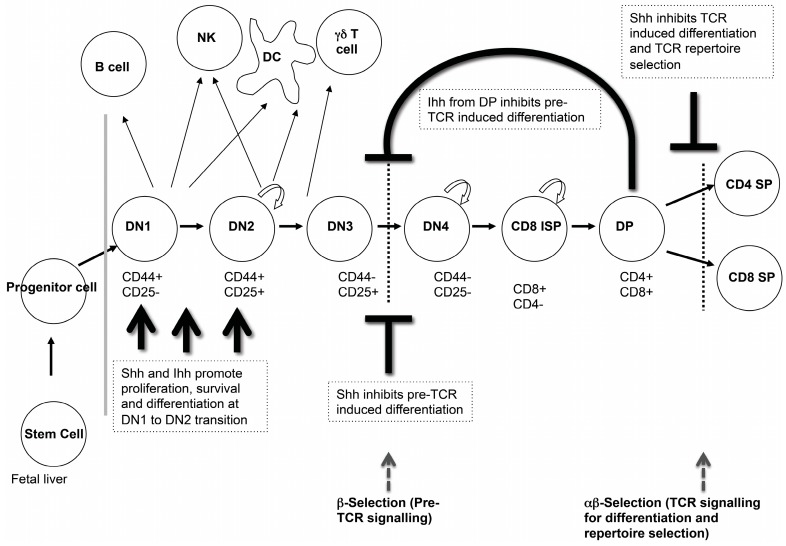
Hh signalling regulates multiple stages of thymocyte development. The cartoon illustrates the different stages of thymocyte differentiation that are regulated by Hh signalling in the embryonic thymus. (TCR: T cell receptor.)

Analysis of T cell development in the foetal thymus of multiple Hh pathway mutants has shown that Hh signalling is an important regulator of T cell development in the embryo, which affects several stages of thymocyte development (Figure 3). We will review the experimental evidence showing that Hh signalling influences three key check-points in embryonic αβT cell development: the transition from DN1 to DN2 cell; pre-TCR induced differentiation to DP cell; and the transition from DP to mature SP cell.

### 3.1. Hedgehog Signalling in DN1 and DN2 Cells

As developing thymocytes differentiate from the earliest DN1 progenitors to express cell surface CD25 and become DN2 cells, they specify to the T cell fate, and initiate recombination of their *TCRβ* gene locus [6]. At this stage, Shh, Gli2 and Gli3 are required for differentiation and expansion of the DN2 population. Thymi from E13.5 *Shh*−/− embryos are much smaller than those of wild type (WT) littermates and have ~10 times fewer thymocytes and a reduction in the DN2 population [19]. Reduced differentiation to DN2 cell has also been observed in E13.5 *Gli2*−/− and *Gli3*−/− thymus [23,25]. In contrast, analysis of Gli1-deficient foetal thymus has shown that Gli1 is dispensable for the DN1 to DN2 transition [24]. In many tissues, Gli3 functions to repress Hh pathway activation, and Gli3-deficiency has the opposite phenotype to Shh-deficiency, so the fact that mutants of both *Shh* and *Gli3* show reduced early thymocyte differentiation might seem surprising. However, the expression pattern of the Gli transcription factors in the DN populations is consistent with the findings from the Gli-mutants in early DN cell development. *Gli2* and *Gli3* are both highly expressed in DN1 and DN2 cells, while expression of *Gli1* in early DN subsets is relatively low (Figure 2).

The role of Ihh in early thymocyte development has also been studied [21]. Analysis of E13.5 *Ihh*−/− thymus shows that deletion of Ihh alone has no influence on development at the DN1 to DN2 transition. However, analysis of the double-mutant *Shh*+/−*Ihh*−/− foetal thymus showed a reduction in the DN2 population, indicating that Shh and Ihh have redundant functions at the DN1 to DN2 transition [21]. 

### 3.2. The Transition from DN to DP Cell and Pre-TCR Signalling

Differentiation from DN3 cell through to DP cell requires successful rearrangement of the *TCRβ* gene locus and formation of the pre-TCR complex [6]. Signalling through the pre-TCR complex results in a complex transcriptional programme, leading to expansion, survival, allelic exclusion of the *TCRβ* gene locus, and differentiation to DP cell [30,31]. In foetal thymocyte development, differentiation from DN3 to DN4 cell seems to be somewhat uncoupled from pre-TCR signal transduction and TCRβ chain expression [32], and thymocytes that have failed to rearrange their *TCRβ* gene locus die at the DN4 stage [32,33].

Hedgehog signalling to DN thymocytes negatively regulates pre-TCR-induced differentiation to DP cell in both mouse and human [22,25,34]. The first evidence for this came from in vitro studies which demonstrated that Hh signalling negatively regulates DN to DP differentiation in foetal thymus organ cultures (FTOCs) that were treated with recombinant (r) Shh [22]. Thymocyte development was arrested at the DN3 stage, whereas FTOCs treated with a neutralizing monoclonal antibody (mAb) against Shh showed increased differentiation from DN to DP cell. Thymocytes from *Recombination-activating gene* *1* (*Rag1*)−/− FTOCs can be induced to differentiate from DN3 to DP cell by treatment with anti-CD3 mAb, which mimics the pre-TCR signal [31,35]. Addition of the anti-Shh mAb to anti-CD3 treated *Rag1*−/− FTOC enhanced thymocyte differentiation to the DP stage, whereas treatment with rShh arrested it, confirming the negative regulatory role of Shh at this developmental stage. 

In the embryo, DP cells first appear on E16.5, allowing observation of the transition from DN to DP population in a synchronized manner. Analysis of the E16.5 *Shh*−/− foetal thymus unexpectedly showed that the transition from DN to DP cell was reduced compared to WT, in contrast to the in vitro studies [19]. However, the E16.5 *Shh*−/− thymus also had increased apoptosis in the DN4 population compared to WT, consistent with increased death of cells that had failed to rearrange the TCRβ locus. This suggested that the requirement for Shh before pre-TCR signalling might impact the production of DP cells on E16.5 [19]. 

In vivo evidence for the negative regulatory role of Hh signalling at this developmental stage came from analysis of foetal thymocyte development in embryos mutant for *Gli2* and *Gli3* [23,25]. From E13.5 to E15.5 *Gli2*−/− thymi were smaller than that of WT littermates, whereas the E17.5 *Gli2*−/− thymus contained over twice as many thymocytes as WT. The expansion of the *Gli2*−/− thymus on the embryonic day after DP cells first appear, together with the expression pattern of Gli2 in the foetal thymus which shows three-fold upregulation in DN4 compared to DN3 cells, suggested that Gli2 plays a negative role in thymocyte expansion and progression to DP after pre-TCR signal transduction during foetal development. This was confirmed in anti-CD3-treated *Rag1*−/−*Gli2*−/− FTOCs, which expanded and differentiated to DP faster than their anti-CD3 treated *Rag1*−/−*Gli2*+/+ counterparts. Interestingly, anti-CD3-treated *Rag1*−/−*Shh*−/− FTOC also differentiated faster than anti-CD3-treated *Rag1*−/−*Shh*+/+ FTOC, confirming that Gli2 is downstream of Shh as a negative regulator of differentiation after pre-TCR signalling [25]. In contrast, analysis of anti-CD3-treated *Rag1*−/− *Gli3*−/− FTOCs revealed a partial arrest at the DN-to-DP stage after pre-TCR signalling [23]. The same result was observed in E16.5 *Gli3*−/− thymus, indicating that Gli3 functions as a transcriptional repressor of the Hh pathway at this developmental stage, and that Gli3 activity is required for normal differentiation from DN to DP cell. 

Given that the Gli transcription factors are expressed by both stromal and thymocyte compartments of the foetal thymus (Figure 2), these experiments with constitutive knockouts did not test whether or not Shh was directly signalling to thymocytes to control their differentiation, or whether the impact on T cell development was an indirect effect due to Hh signalling to TEC or another stromal cell population. Therefore, the impact of Hh-mediated transcription in developing thymocytes on their differentiation and function was investigated by the production of transgenic (tg) mice which over-expressed activator (Gli2A-tg (Gli2ΔN2-tg)), or repressor (Gli2R-tg (Gli2ΔC2-tg)) forms of Gli2 in T lineage cells only (driven by the *lck* promoter) [36,37]. Anti-CD3-treated *Rag1*−/− Gli2A-tg FTOC differentiated more slowly to DP than their non-transgenic counterparts, whereas anti-CD3-treated *Rag1*−/−Gli2R-tg FTOC—in which physiological Hh-mediated transcription is repressed—differentiated more quickly [25]. Thus, Hh pathway activation in developing thymocytes negatively regulates pre-TCR-mediated differentiation to DP cell.

### 3.3. Ihh at the Transition from DN to DP Cell and Its Role in Homeostasis and the Control of DP Cell Number

Ihh is produced by both the foetal thymus stroma and developing thymocytes with a six-fold higher expression in the DP population compared to DN cells and stroma, which show similar *Ihh* expression levels [21] (Figure 2). The DP population expresses low levels of the target gene *Gli1*, suggesting that Ihh is not signalling to the DP population in an autocrine manner, but feeding back to signal to the DN3 population, which express the highest levels of *Gli1*. 

In the E16.5 *Ihh*−/− embryo, both thymocyte numbers and the proportion of DP cells were reduced, indicating that Ihh promotes T cell development. Interestingly, however, the E16.5 *Ihh*+/− thymus contained 2.4 times more DP cells than WT, implying that Ihh also negatively regulates thymocyte development. *Ihh*+/+ *Rag1*−/− FTOCs contained more cells than their *Ihh*+/− *Rag1*−/− counterparts, but after anti-CD3 treatment for 5 days, the *Ihh*+/− *Rag1*−/− FTOCs differentiated faster than the *Ihh*+/+ *Rag1*−/− FTOC, confirming that Ihh promotes early DN thymocyte development before the pre-TCR signal, but negatively regulates the pre-TCR-induced transition to the DP stage. 

In the *Ihh*+/− E16.5 thymus, the sorted CD25^+^ DN population contained significantly more cells in cell cycle (S/G2+M) than its WT counterpart. Taken together, these data indicated that Ihh produced by the DP population feeds back to negatively regulate the differentiation and expansion of the DN3 population after pre-TCR signal transduction. Thus, as the total amount of Ihh protein present in the thymus largely depends on the size of the DP population, this negative feedback loop may be thought of as a counting system to regulate thymocyte number and maintain thymocyte homeostasis [21]. 

### 3.4. Hh Signalling in TCR Repertoire Selection and the Transition from DP to SP Cell

Hh signalling also plays an important role during the maturation of DP to CD4SP and CD8SP cell in the embryonic thymus. Maturation from DP to SP cell occurs following successful rearrangement of the *TCRα* gene locus, and requires TCR signalling: positive selection ensures appropriate MHC-restriction of SP cells, followed by negative selection of potentially self-reactive clones [3,6]. Many models have been proposed to describe how DP thymocytes commit to the CD4 and CD8 lineages, and to explain how positive selection ensures that selected CD4 and CD8 SP populations express TCR appropriately restricted by MHCII and MHCI, respectively [38]. Strength and duration of the TCR signal that a developing cell receives broadly determine its fate, with the strongest signals leading to negative selection, usually at the SP stage in the medulla (of TCR recognizing self-antigens)—intermediate signals leading to positive selection, and weaker signals or lack of TCR signalling leading to cell death by neglect. For DP thymocytes undergoing positive selection, TCR signal strength and duration again influence CD4 and CD8 fate decision, with those cells receiving stronger TCR signals tending to be biased towards the CD4 fate, and weaker/more transient signals tending to favour differentiation to CD8 SP. TCR signal strength and duration are dependent on the avidity of the TCR for its ligand (and therefore on the TCR sequence), but may also be modulated by other factors, such as coreceptor signalling, intracellular or extracellular influences on TCR signal transduction, and integration of the transcriptional outcome of TCR signalling with the transcriptional context of the cell.

**Figure 4:**
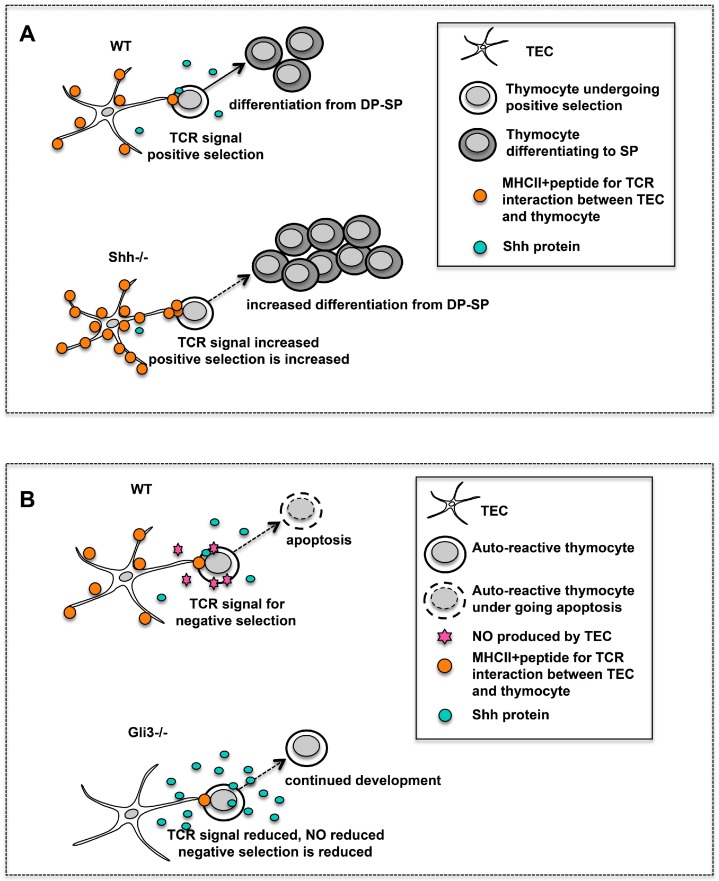
Hh signalling at the transition from DP to SP thymocyte. (**A**) The cartoon illustrates the influence of Shh on positive selection. Positive selection is increased in the *Shh*−/− thymus and the proportion of CD4SP cells is increased. Cell surface expression of major histocompatibility complex class II (MHCII) is increased on *Shh*−/− TEC compared to wild type (WT) TEC. (**B**) The influence of Gli3 on negative selection. In the *Gli3*−/− thymus MHCII expression is decreased in TEC and nitric oxide (NO) activity is decreased. This may allow SP thymocytes to escape from negative selection.

Analysis of embryonic thymus from *Shh*−/−, *Gli1*−/−, *Gli2*−/−, Gli2A-transgenic (tg) and Gli2R-tg, and in vitro FTOC experiments have all shown that Shh negatively regulates the DP to SP transition, most likely by lowering TCR signal strength (see Figure 4) [24,26,36]. 

The *Shh*−/− embryonic thymus is smaller than WT but has a higher SP:DP ratio, reflecting an accelerated rate of differentiation from DP to SP [36]. *Shh*−/− FTOCs had a greater proportion of mature CD4SP cells and an increased CD4SP:CD8SP ratio than WT (Figure 4A). Likewise, *Gli2*−/− and *Gli1*−/− FTOCs showed increased differentiation from DP to SP cells and increased CD4SP:CD8SP ratio [24]. The increased differentiation towards the CD4SP lineage might reflect an increase in TCR signal strength, and both positive and negative selection of transgenic TCR were increased in *Shh*−/−, *Gli2*−/−, and *Gli1*−/− FTOC compared to control [24,36]. Analysis of the Gli2A-tg and Gli2R-tg embryonic thymus confirmed that physiological Hh-mediated transcription in thymocytes negatively regulates the DP-to-SP transition and TCR repertoire selection [26].

Cell surface CD5 intensity is an indicator of TCR signal strength on thymocytes, with higher CD5 expression reflecting a stronger TCR signal and vice versa [39,40]. Gli2R-tg FTOCs, in which physiological Hh-mediated transcription is inhibited, showed increased differentiation to CD4SP, higher CD5 expression on the CD4SP population, and a higher CD4SP:CD8SP ratio than WT [26]. Doubling the Gli2R-transgene copy number further increased the proportion of CD4SP cells, their CD5 cell surface expression, and the CD4SP:CD8SP ratio [26]. Interestingly, these dose-dependent phenotypic changes were reversed with rShh treatment of WT FTOC. Decreased cell surface CD5 expression and a lower CD4SP:CD8SP ratio were observed when WT FTOC were treated with rShh [36]. Therefore, an increased level of Shh signalling appeared to give rise to weaker TCR signal strength, leading to reduced selection from DP to CD4SP; whereas selection to the CD8 lineage, which is believed to require lower strength and duration of TCR signal transduction, was less affected. 

Gata3 is a key regulator of CD4 lineage commitment, and strong TCR signals increase expression of Gata3, which drives differentiation towards the CD4 lineage [41]. Interestingly, treatment of WT FTOCs with rShh decreased the proportion of Gata3-expressing DP and SP cells, while attenuating Shh signalling by treatment with the neutralising anti-Shh mAb increased the intensity and expression of Gata3 on the DP and SP4 populations [26]. Thus, the influence of modulation of Shh signalling on Gata3 expression is consistent with the effect of Shh on CD4/CD8 lineage commitment, and with its influence on TCR signal strength [36,42].

TCR signal strength is a crucial determinant of the TCR repertoire and CD4/8 lineage commitment and the influence of Shh on TCR signal strength may thus alter both of these: Since Shh-expressing TECs are scattered around the medulla and the cortico-medullary junction, their influence on TCR signal strength and the outcome of TCR ligation (i.e., positive or negative selection and CD4/8 lineage decision) for each cell is dependent on the location of the cell relative to the source of Shh. It is therefore possible that Shh-expressing TEC may have specialised functions in the thymus, such as induction of positive selection or commitment to the CD8 lineage. 

### 3.5. Gli3 Regulates Nos2 Expression during Negative Selection

Analysis of the *Gli3* mutant thymus showed that Gli3 acts as a repressor of Hh signalling in the foetal thymus and that Gli3 mutation may also influence TCR repertoire selection in developing thymocytes [23,27]. These experiments were carried out using constitutive Gli3 knock-outs, so that the lineage-specific requirements for Gli3 are not known, and both the *Gli3*−/− foetal thymus stroma and foetal thymocytes had increased expression of the transcriptional target of Hh-signalling, *Gli1* [24,27]. Treatment of *Gli3*−/−FTOC with a neutralising anti-Shh mAb reduced *Gli1* expression in the stroma [27], demonstrating that the increase in *Gli1* expression was a result of increased Hh signalling. 

PCR array analysis of gene expression in the *Gli3*−/− foetal thymus stroma compared to WT identified Gli3-target genes in the stroma, and revealed that *Nos2* is a Gli3 target gene, which was significantly down regulated in the *Gli3*−/− and *Gli3*+/− stroma [27]. *Nos2* expression recovered to WT levels when *Gli3*+/− FTOC was treated with an anti-Hh neutralising mAb, but was not affected when *Gli3*−/− FTOC were treated with an anti-Hh mAb, suggesting that *Nos2* required Gli3 for its physiological levels of expression (most likely through Gli3R’s repression of an intermediate transcriptional repressor of *Nos2*) [27]. 

Nos2 is induced in the thymic stroma during negative selection. It causes the production of nitric oxide (NO), a potent pro-apoptotic agent, which promotes apoptosis of autoreactive thymocytes [43]. Treatment of WT and *Gli3*−/− FTOC with anti-CD3 mAb to mimic negative selection, showed that the *Gli3*−/− DP cells had decreased intracellular active caspase-3 expression, correlating with a lower level of apoptosis compared to WT. This suggests that negative selection-related apoptosis of DP thymocytes is attenuated in the *Gli3*−/− thymus and that this may be due to reduced *Nos2* expression in the thymic stroma. (Figure 4B). It is likely that this would lead to a change in the TCR repertoire and survival of autoreactive thymocytes in the embryonic thymus. Indeed, negative selection of an MHCI-restricted transgenic TCR is reduced in the adult *Gli3*+/− thymus [27], although the impact of Gli3-mutation on negative selection of a transgenic TCR has not been investigated in the embryonic thymus.

## 4. Hedgehog Signalling in Foetal Thymic Epithelial Cell (TEC) Development and Function

The role of the Hh pathway in embryonic TEC development has been less extensively investigated than its function in T cell development, although Shh is involved early in thymus organogenesis during patterning of the pharyngeal pouches [44]. In this section, we will briefly discuss the early role of Shh in pharyngeal patterning and thymus organogenesis before reviewing recent experiments demonstrating that Hh signalling is required for normal TEC development in the embryonic thymus during the last week of gestation. 

Shh is involved in the formation and patterning of the entire pharyngeal apparatus and affects the third pouch and the thymic anlage [44]. In *Shh*−/− embryos, the development of the third pharyngeal pouch is defective and the thymic rudiment fails to bud off entirely from the pharyngeal endoderm [45]. However, once the *Shh*−/− thymus is formed, although it is small, it is capable of supporting T cell development; however, there are defects in normal TEC development [19,21]. 

There are two types of TEC, which are derived from a common progenitor, and which have distinct functions, localisations within the thymus, and cell surface markers [2,4,5,46]. Cortical(c) TECs provide DL4 for T cell fate specification, and present MHC+peptide ligands for positive selection. These cTECs are defined as EpCam1+, CD205+, Ly51+ and MHCII+, and express genes for antigen presentation, including *Cathepsin-L*, *Prss16,* and *β5t*. Medullary(m) TECs are specialised for induction of negative selection, are defined as cell surface EpCam1+, CD40+, CD205−, Ly51− and MHCII+, and bind the lectin UEA-1. They express the *Aire* gene and *Cathepsin-S*, facilitating the expression and presentation of Tissue Restricted Antigens (TRA) for induction of tolerance. During embryonic development, the cell surface markers CD40 and CD205 can be used to map TEC development. CD40^low^CD205^low^ progenitor cells gain cell surface CD40 and CD205 expression, resulting in transitional progenitors which have the potential to differentiate into the two mature populations: cTEC (CD40^int^CD205^high^Ly51+) and mTEC (CD40^high^CD205^low^UEA-1+) [9,10]. 

Embryonic TECs express components of the Hh signalling pathway as well as the genes encoding the Hh ligands (Figure 2). Therefore, to investigate if TECs are transducing Hh signals in vivo in the embryonic thymus, Gli Binding Site (GBS)-Green Fluorescent Protein (GFP) transgenic embryos—which report Hh-mediated transcription by expression of GFP—were analysed [27]. Hh pathway activation was observed in the TEC populations of embryos from E14.5 through to birth (neonate). The greatest intensity of GFP-fluorescence was observed on E14.5, and fluorescence was seen in the CD40^low^CD205^low^ progenitor cells, which made up about one third of TEC, and in the emerging CD40^int^CD205^high^ and CD40^high^CD205^low^ populations. On E18.5, higher expression was observed in cells that had specified to the mTEC lineage. At birth, the CD40^low^CD205^low^ progenitor population made up less than 1% of all TEC, but both the mature cTEC and mTEC populations contained about 20% of cells that expressed GFP [29].

**Figure 5:**
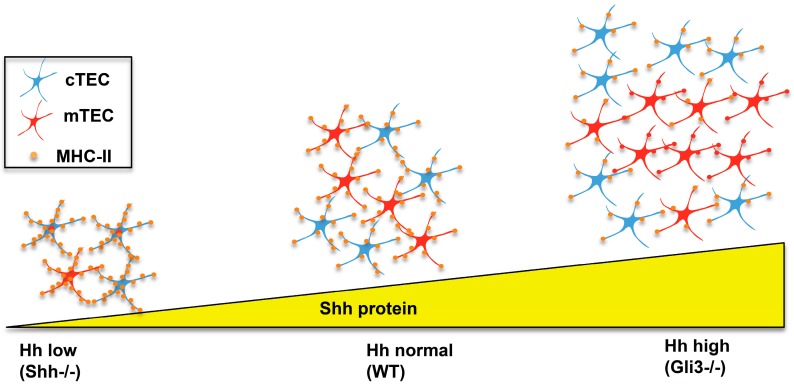
Impact of Shh-deficiency and Gli3-deficiency on TEC differentiation and function. In the *Shh*−/− embryonic thymus, Hh protein levels are low, there are fewer TEC, and the ratio of mTEC (shown in red) to cTEC (shown in blue) is decreased, but expression of MHCII is increased on individual TEC, compared to WT. In the *Gli3*−/− embryonic thymus—which has increased levels of Hh pathway activation—the opposite phenotype is seen. TEC numbers are increased, but MHCII expression on individual TEC is reduced compared to WT.

Analysis of the *Shh*−/− foetal thymus has recently shown that Shh is required for normal TEC development and the regulation of mTEC/cTEC lineage choice [29]. In the *Shh*−/− foetal thymus, there was a reduction in thymus size, overall cell number, and number of thymocytes and of TECs (CD45−EpCam1+) compared to WT littermates [19,21,29]. The numbers of both mature cTECs (CD45−EpCam1+Ly51+UEA1−) and mTECs (CD45−EpCam1+Ly51−UEA1+) were reduced in *Shh*−/− compared to WT, but the mTEC lineage was particularly affected. The proportion of mTECs generated in *Shh*−/− FTOC fell from 27.4% to 13.2%, compared to WT FTOC, with a concomitant increase in the proportion of mature cTECs [29]. Interestingly, although the number of TECs was reduced in the *Shh*−/− FTOC, cell surface expression of MHCII was increased in both cTEC and mTEC (Figure 5) [29]. Expression of MHCII is essential on cTECs to provide ligands for positive selection for differentiation from DP to CD4SP, and on mTECs for negative selection of MHCII-restricted thymocytes. The increase in MHCII expression on cTECs and mTECs in the *Shh*−/− foetal thymus could therefore potentially influence the outcome of positive and negative selection of thymocytes (Figure 3, Figure 4 and Figure 5) [29]. Although there are overall fewer TECs in the Shh-deficient thymus, on each individual TEC, more MHCII-plus-peptide complexes for positive and negative selection will be available to developing thymocytes, and so it is possible that this may affect the outcome of TCR repertoire selection to the CD4 T cell lineage by changing MHCII-restricted TCR antigen dwell time or avidity [47] (see Figure 4 and Figure 5). 

The influence of Shh on foetal TEC development was confirmed when WT FTOC were treated with recombinant Hhip, a Hh-binding protein that neutralizes endogenous Hh proteins in the cultures. Hhip-treatment led to an overall decrease in TEC cell numbers and a significant decrease in the proportion of mature mTECs. The proportion of mTECs that expressed Aire protein was also reduced, but there was an increase in cell surface MHC-II on the mTEC population [27].

The opposite was observed in *Gli3*−/− foetal thymus (Figure 5). There was an increase in overall TEC numbers but a decrease in cell surface expression of MHC-II in both cTEC and mTEC populations (Figure 4B and Figure 5) [29]. This is consistent with Gli3 acting as a suppressor of *Shh* in the foetal thymus [27]. 

## 5. Conclusions and Future Directions

Hedgehog signalling influences multiple stages of T cell development, and also regulates foetal TEC differentiation (Figure 3 and Figure 5). Very few pathways or secreted signalling molecules have been identified that regulate foetal TEC development or cTEC/mTEC lineage choice, so it will also be important to identify the mechanisms of Shh’s regulation of TEC differentiation in the embryonic thymus. It will be interesting to identify Hh target genes in different subsets of TEC, and test the impact of mutation of these genes on TEC development and function, and on the ability of TEC to support T cell development.

Hh signalling influences three key check-points in embryonic αβT cell development: the transition from DN1 to DN2 cell; pre-TCR-induced differentiation to DP cell; and the transition from DP to mature SP cell. The different mechanisms that account for these different developmental-stage specific outcomes of Hh pathway activation in developing T cells are as yet unknown, and Hh target genes have not been identified in thymocyte subsets.

In the future, it will also be important to investigate the interactions between Hh signalling and other pathways that interact with Hh signalling in other tissues, and also regulate foetal thymocyte development—such as BMP2/4 signalling [48,49,50]. BMP2/4 signalling is also involved in early thymus organogenesis and is required for early TEC development, so it will also be interesting to assess interactions between BMP and Shh signalling in TEC development [51,52].
